# Impact of proton beam availability on patient treatment schedule in radiation oncology

**DOI:** 10.1120/jacmp.v13i6.3968

**Published:** 2012-11-08

**Authors:** Eric D. Miller, Vladimir Derenchuk, Indra J. Das, Peter A. S. Johnstone

**Affiliations:** ^1^ Indiana University Health Proton Therapy Center, Bloomington IN & Department of Radiation Oncology Indiana University School of Medicine Indianapolis IN 46202 USA; ^2^ Indiana University Cyclotron Operations Bloomington IN 47408 USA

**Keywords:** proton therapy, quality, beam availability, health services research, uptime

## Abstract

Proton beam therapy offers unique physical properties with potential for reduced toxicity and better patient care. There is an increased interest in radiation oncology centers to acquire proton therapy capabilities. The operation of a proton treatment center is quite different than a photon‐based clinic because of the more complex technology involved, as well as the single proton beam source serving multiple treatment rooms with no backup source available. There is limited published data which investigates metrics that can be used to determine the performance of a proton facility. The purpose of this study is to evaluate performance metrics of Indiana University Cyclotron Operations (IUCO), including availability, mean time between failures, and mean time to repair, and to determine how changes in these metrics impact patient treatments. We utilized a computerized maintenance management system to log all downtime occurrences and servicing operations for the facility. These data were then used to calculate the availability as well as the mean time between failures and mean time to repair. Impact on patient treatments was determined by analyzing delayed and missed treatments, which were recorded in an electronic medical record and database maintained by the therapists. The availability of the IUCO proton beam has been increasing since beginning of operation in 2003 and averaged 96.9% for 2009 through 2011. The mean time between failures and mean time to repair were also determined and correlated with improvements in the maintenance and operating procedures of the facility, as well as environmental factors. It was found that events less than 15 minutes in duration have minimal impact on treatment delays, while events lasting longer than one hour may result in missed treatments. The availability of the proton beam was more closely correlated with delayed than with missed treatments, demonstrating the utility and limitations of the availability metric. In conclusion, we suggest that the availability metric and other performance parameters, such as the mean time between failures and the mean time to repair, should be used in combination with downtime impact on patient treatments in order to adequately evaluate the operational success of a proton therapy facility.

PACS number: 87.55.‐x

## I. INTRODUCTION

Proton beam therapy provides a unique opportunity for better patient care by delivering uniform dose to the tumor with minimal integral dose to the body, thus possibly reducing complications associated with radiation treatments.^1^, ^2^ There has been a recent rush for radiation oncology centers to acquire particle beam therapy capabilities; however, the technology is much more complex.

Running a photon clinic is fairly straightforward. Most centers have more than one linear accelerator (linac), so if one becomes nonfunctional, patients can be moved to another unit, and treatment is unaffected. Short of catastrophic loss (e.g., wave guides, target, klystron, magnetron), linac maintenance contracts allow for rapid repair in most localities. Many centers have repair personnel on staff or are covered by the larger maintenance teams of accelerator vendors for an annual maintenance fee. The uptime of even linear accelerator‐based oncology departments is within a wide range from 92%–98%.^3^, ^4^ Dawson and Gribble^(4)^ showed that in‐house engineering staff could improve uptime from 92% to 97.2% for a simple 6 MV beam. Despite the increasing complexity of modern accelerators with multileaf collimators, on‐board imaging, variable dose rate (100–800 MU/min) and high degree of mechanical precision as required by TG‐142,^(5)^ the technology is sufficiently reliable that the uptime of modern machines is speculated to be up to 98% and decreasing to as low as 84% for installations over 10 years old.^6^, ^7^ Preventive maintenance (PM) on linacs is well defined and based on years of experience with hundreds or thousands of identical units in the field. Because of technological advances, health organizations are motivated to replace linacs long before the end of their useful life.

Cyclotron or synchrotron‐based proton treatment centers are a stark contrast to linac‐based clinics. The PM plan is of enormous importance because the useful life of these units is anticipated to be several decades. Operating a proton center requires a thorough weekly PM plan backed by highly trained service personnel with a comprehensive supply of spare parts. In no case in the United States does a colocated backup unit exist should the primary fail. Further, simply moving patients from one treatment room to another is only possible if the problem is downstream of the beam delivery system. Given the technology involved and lack of a backup unit for the accelerator, the schedule for the clinic's entire case load could be impacted catastrophically. If the beam is restored in a timely fashion, it can simply be a scheduling inconvenience for the patients and staff as treatments can continue later into the evening. In the worst case scenario, treatments are missed, which adversely impacts the patients scheduled to be treated and also new patients waiting to begin their treatment course.

One metric that proton therapy centers use to determine performance and efficiency of their treatment facilities is beam availability $\left(A_{v}\right)$.^(8)^ $A_{v}$ is broadly defined as the fraction of time the treatment room is online and can be used to treat patients. $A_{v}$ is frequently used as a standard metric compared across institutions. However, centers vary in calculating $A_{v}$. In a recent study from Suzuki et al.,^(8)^ only events resulting in treatment breaks longer than 15 minutes were included as part of the downtime calculation. Suzuki et al. reported an average yearly $A_{v}$ of 97% from June 2007 to August 2010 for the MD Anderson Proton Therapy Center. Loma Linda University Medical Center (LLUMC) calculated $A_{v}$ in a different manner.^(9)^ The uptime and, thus, the $A_{v}$ was based on the number of treatments missed due to equipment failure, not accounting for the actual length of time that the facility can be used to deliver treatments. In 2009, LLUMC reported an average $A_{v}$ of 98.8%.^(9)^

Limited data on beam $A_{v}$ have been reported for cyclotron or synchrotron‐based particle beam therapy units, possibly due to the limited number of centers. Similarly, to the best of our knowledge, there have not been any reports investigating how $A_{v}$ relates to the patient treatment schedule. The purpose of this study was to evaluate the $A_{v}$ as well as additional performance metrics used at Indiana University Cyclotron Operations (IUCO), the mean time between failures (MTBF), and mean time to recovery (MTTR). We also determined how the length of downtime events impact patient treatments. Finally, we investigated how $A_{v}$ correlates with delayed and missed patient treatments.

## II. MATERIALS AND METHODS

### A. Beam delivery system

IUCO was formerly known as the IU Cyclotron Facility (IUCF). Responsibility for the clinical care of patients using the proton beam falls to Indiana University (IU) Health Proton Therapy Center (IUHPTC). Responsibility for the design, fabrication, maintenance, and upgrade of the IU proton treatment system falls to IUCO, which is a FDA‐registered medical device manufacturer. IUCO has approximately 30 employees dedicated to operating and servicing the proton therapy equipment. There are an additional 35 employees who make up the physics and radiation effects research groups, the in‐house engineering department that designs upgrades to improve reliability and functionality and provides hardware, software, and electronics support for the treatment systems, and a machine shop that manufactures all patient‐specific devices for IUHPTC. The IU proton treatment facility consists of three treatment rooms supplied by a common proton accelerator and beam delivery system. One treatment system has a fixed horizontal beam line, while the other two feature a 360° rotating gantry system each with a uniform scanning beam nozzle. The 208.4 MeV beam is generated by a sequence of a 750 keV radio frequency quadrupole linear accelerator,^(10)^ a 15 MeV cyclotron, and a 208.4 MeV cyclotron.^(11)^ The cyclotrons are operated continuously, delivering beam to a dump which is diverted to the treatment rooms or research users on request using a fast (millisecond time scale) kicker magnet at the entrance of each room. From the kicker magnet, the proton beam is transported to the proton nozzle via an energy selection beam line containing an energy degrader which adjusts the proton range between 5 cm and 27 cm (beam energy from 65 MeV to 208.4 MeV). Commissioning of the accelerators and fixed horizontal beam room began in 2003, with the first patient treatments in April 2004. From 2004 to 2006, only the fixed beam line room was available to treat patients. In 2007, the first gantry room went into service and, in 2008, a second gantry room was available for treating patients. The beam delivery system has been described in more detail previously.^(12)^

When the center first opened in 2004, patient treatments were administered for 8 hours per day and 5 days per week. When the gantries went into service, the number of patients increased and the hours increased to 12 hours per day, 5 days per week. For the past year, the patient numbers have continued to increase, and the center is currently treating patients 15 hours per day, 5 days per week. Quality assurance testing occurs each morning prior to the start of patient treatments, as well as each weeknight after all treatments have been completed. Preventative maintenance of the system is performed primarily on weekends.

### B. Quality metrics

At IUCO/IUHPTC, $A_{v}$ is a measure of the fraction of time all three treatment rooms are available for scheduled use. Scheduled use includes: patient treatments, research, commissioning of new equipment, and any quality assurance testing. We also include any time when the schedule is extended due to unforeseen circumstances. $A_{v}$ is represented by Eq. (1).

(1) Availability=100×Uptime/(Uptime+Downtime)

where *uptime* and *downtime* are summed for all three treatment rooms. Downtime includes any interruptions where the proton beam is requested for research, testing, or treatment, but cannot be delivered regardless of the cause.

Other metrics used by IUCO are the MTBF and the MTTR. The MTBF is a time function of the reliability of the system and represents the average time between events which cause downtime of the system. The MTTR is the average time required to restore equipment to service after a downtime event has occurred. It includes time to diagnose the problem, perform the necessary repair or replacement, perform quality checks, and finally validate the system so that patient treatments may continue.

### C. Data acquisition

IUCO utilizes a computerized maintenance management system (CMMS) to log all servicing operations such as preventive maintenance, improvements and upgrades, and any unscheduled or emergency servicing actions. Failures of equipment and abnormal operating events are captured in a nonconformance reporting system, tracked and analyzed by IUCO servicing and engineering staff. Independent of the CMMS, Proton Therapy System operation is captured in an electronic logbook and is used to identify time and duration of all events impacting the schedule and clinic use of the beam. All downtime occurrences are logged regardless of their duration. For each downtime event, IUHPTC monitors and records the number of patients whose treatments were impacted using both an Excel (Microsoft Corp., Redmond, WA) spreadsheet and Multi‐ACCESS software (IMPAC Medical Systems, Inc., Mountain View, CA). We define impacted treatments as: 1) patients whose treatment was delayed but still delivered on the planned day of treatment; 2) patients whose treatment was missed as a result of the downtime event.

**Figure 4:**
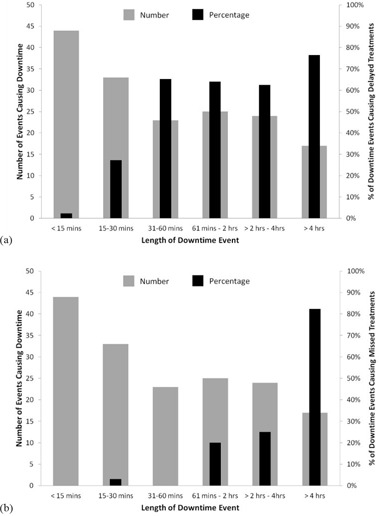
The number of downtime events and the corresponding percentage of those events which caused treatment delays (a) and missed treatments (b) stratified by the length of the downtime event for calendar year 2011. The number of events is indicated by the gray bars and includes all events where any component of the system was down. The percentage of those events which had an impact on patient treatments is indicated by the black bars.

**Figure 5:**
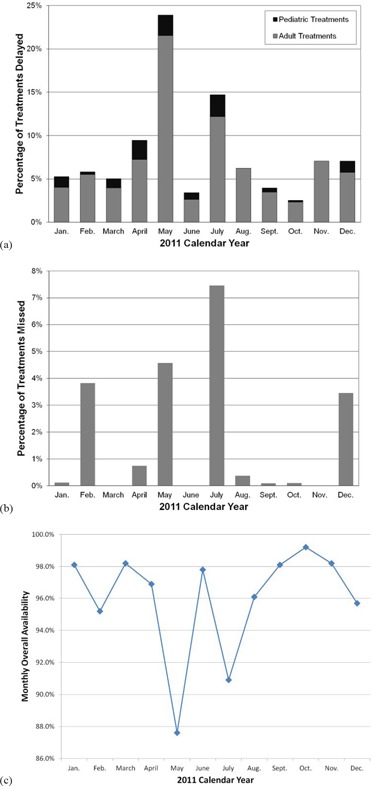
The percentage of treatments (a) which were delayed by month for calendar year 2011 (the data is separated into pediatric treatments shown in black and adult treatments shown in gray); the percentage of missed treatments (b) by month for calendar year 2011; and the monthly overall availability (c) for calendar year 2011.

**Figure 6:**
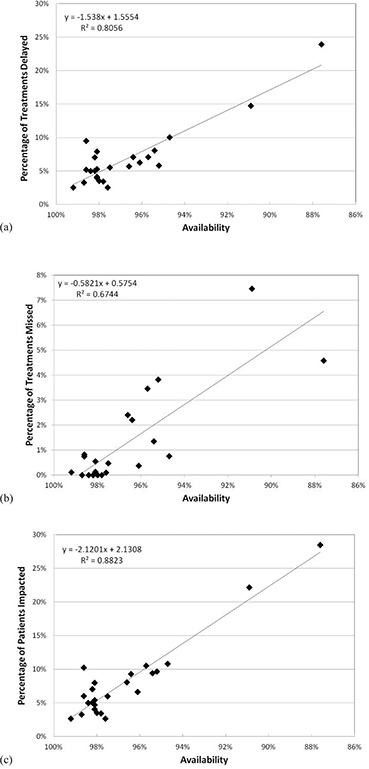
The percentage of treatments delayed (a), the percentage of treatments missed (b), and the percentage of patients impacted (missed or delayed treatment) (c) are plotted as a function of the overall availability for each month from January 2010 through December 2011. The line fitted to the data was obtained using least squares linear regression.

**Figure 1:**
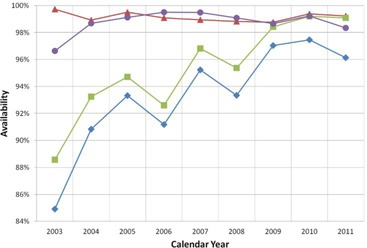
Availability for calendar year 2003 to 2011. Data points correspond to overall availability (♦), availability for the treatment room systems from the kicker magnet through the nozzle and patient positioning system (A), the cyclotron and beamlines alone (■), and external issues (•) which includes downtime related to power failures, weather related events, user errors, and failure of the X‐ray system.

**Figure 2:**
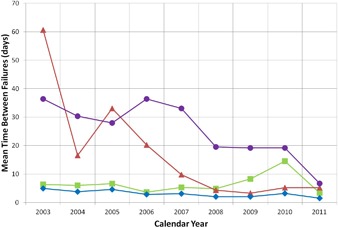
Mean time between failures (MTBF) from 2003 to 2011. Data points correspond to the overall system (♦), the treatment room systems (A), the cyclotron (■), and external issues (•) such as power failures and weather related events.

**Figure 3:**
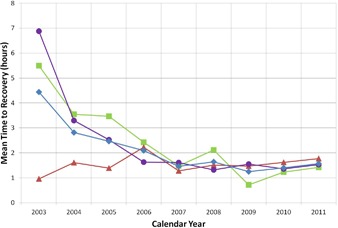
Mean time to recovery (MTTR) from 2003 to 2011. Data points correspond to the overall system (♦), the treatment room systems (▲), the cyclotron (■), and external issues (•) such as power failures and weather related events.

The annual $A_{v}$, as well as the MTBF and MTTR reported in this article (Figs. 1–3), were determined using data obtained by CMMS from calendar years 2003 to 2011. The relationship between the number and duration of downtime events and patient treatments (Fig. 4), as well as the percentage of missed and delayed treatments per month and monthly $A_{v}$ (Fig. 5), was determined by correlating the event log from CMMS with the patient log maintained by IUHPTC for the 2011 calendar year. The same method was used to determine the correlation between $A_{v}$ and patient treatments (Fig. 6) for the time period between January 2010 and December 2011.

## III. RESULTS

### A. Availability

The $A_{v}$ since proton beam operation began in 2003 is shown in Fig. 1. The $A_{v}$ related strictly to the treatment room systems alone has averaged at approximately 99% since startup in 2003. The contribution to downtime from external factors is mainly driven by weather and power failure events and fluctuates between 0.5% and 2% each year since 2004. The $A_{v}$ of the cyclotron systems has remained above 95% since 2008 and its contribution to downtime has recently stabilized to 1%–2% each year. This increase in cyclotron and, thus, overall $A_{v}$, is evidenced by an improvement in the MTBF and MTTR, which can be attributed to redesigning or replacing unreliable accelerator equipment, changes in maintenance and operating procedures, stocking critical spare parts, and improving technical skill levels in our staff.

A measure of PM and operating procedures is illustrated by the MTBF shown in Fig. 2. Most maintenance and accelerator shutdowns occur on weekends when major pieces of equipment are turned off, causing stress on the equipment. Thus, an average of 5 to 10 days between failures for accelerator hardware as seen in the cyclotron curve in Fig. 2 is directly related to weekend maintenance. The increase in MTBF in 2010 for the cyclotron curve is a result of modifications of the system in 2009 to increase reliability. Another cause of accelerator failures is related to power quality events, which cause stress on the equipment and increase failure rates. There were numerous issues with electrical power in 2011, as seen by the drop in the curve representing external issues including power failures. The MTBF corresponding to issues with power failures is directly related to local weather patterns with numerous episodes of severe weather occurring in 2011. For the treatment room systems, the sudden drop in MTBF in 2004 was due to the initiation of patient treatments. It has steadily decreased from 2005 to 2009 corresponding to the transition from the single fixed beam line room to two gantry treatment rooms and keeping pace with the increase in number of patients being treated. With more machinery and increased complexity, issues occurred more often during this time period. Since 2009, the MTBF for the treatment room systems has been slowly improving, due to design changes of equipment that began to fail due to heavy use.

Figure 3 shows the MTTR, which is an indicator of how quickly the system can be brought back online to resume patient treatments. The MTTR for the cyclotron has decreased since 2003 due to the redesign of critical components so that they are easier to repair and an improving PM program that detects issues before they become catastrophic failures.^(13)^ There has also been improvement in restoring the system to operation after a power failure. The MTTR for the treatment room systems has remained fairly constant over the past several years, remaining between 1 and 2 hours despite steadily increasing use of the equipment. The slight increase from 2010 to 2011 for all four curves corresponds to severe power failures in May 2011 that required a longer recovery time.

### B. Patient impact

The success of day‐to‐day operation of a proton therapy center can also be judged by the patient impact of unscheduled events and downtime. We evaluated the percentage of days per month in calendar year 2011 where treatments were either delayed or missed due to downtime events. Treatments were affected due to downtime an average of 27.5% of the days or a little less than once every three days. In months with bad weather, such as May 2011, patients were impacted nearly every day. In January 2011, the treatment schedule was impacted only twice.

While delays of several hours can be catastrophic for the clinic and result in missed treatments, frequent 10‐minute delays can also be troublesome, particularly if no extra time is scheduled between patients. Figure 4 shows the distribution of all events sorted by length of downtime for calendar year 2011 separated into the percentage of events causing delayed treatments (a) and missed treatments (b). The majority of events were less than 15 minutes and between 15 and 30 minutes in length. The category representing events lasting longer than 4 hours was the smallest. To determine the impact of the length of the downtime event on patient treatments, each downtime event was correlated with the clinical database to determine if treatments were delayed or missed due to the event. As seen in (Fig. 4a), longer downtime events are more likely to delay patient treatments. Fewer than 5% of events lasting less than 15 minutes delayed patient treatments. In every other category, patient treatments were delayed at least 25% of the time. Events that last less than 15 minutes do not result in treatment delays because the majority of these events go unnoticed by the clinic. For example, many of the short cyclotron downtime events occur between patient treatments or when beam is not required and will have no effect on the patient schedule. Whether or not a downtime event results in a delay also depends on other factors including which piece of equipment is affected and how much time is scheduled for each treatment. Events lasting longer than 4 hours resulted in delayed treatments in nearly 80% of cases. Less than 100% of treatments are affected by such a lengthy delay because some of the downtime events occurred during quality assurance time and not when patients were being treated. These data demonstrate that shorter events can be overcome relatively easily, while longer events are more likely to result in treatment delays. (Figure 4b) demonstrates that events less than 60 minutes in duration are not likely to cause missed treatments, in contrast to events lasting longer than one hour where treatments are missed. This result indicates that events lasting longer than one hour are the threshold for moving from treatment delays to missed fractions in a patient's course of treatment.

To determine how $A_{v}$ impacts patient treatments, we correlated both delayed and missed treatments with the monthly $A_{v}$ for the 2011 calendar year (Fig. 5). (Figure 5a) shows the percentage of treatments which were delayed stratified into adult and pediatric treatments, respectively. Because of the high priority placed on the pediatric population, the percentage of delayed pediatric treatments was much less than the adult patients with the pediatric treatments representing less than 3% of rescheduled treatments for all months of the 2011 calendar year. The months with the most pediatric treatment delays were April, May, and July. During May and July, there were an increased number of weather events including power outages and days during which very high temperature and humidity combined exceeded the facility's cooling capacity and resulted in a correspondingly large number of treatment delays for both adult and pediatric patients. The increased percentage of delayed pediatric treatments observed in April corresponds to several power outages which occurred during the morning treatment sessions. (Figure 5b) shows the percentage of treatments missed during the 2011 calendar year. Similar to (Fig. 5a), May and July had the largest number of missed treatments due to weather events. There were also an increased number of missed treatments observed in February and December. The missed treatments observed in February were due to several cyclotron‐related issues that occurred during the afternoon treatment sessions and resulted in patient treatments being cancelled for the rest of the day. The increase in December corresponds to one major cyclotron event that resulted in no treatments being administered for that day. The overall $A_{v}$ for each month of the 2011 calendar year is shown in (Fig. 5c). May and July had the lowest $A_{v}$ for the year, 88% and 91%, respectively, corresponding to the increased weather events during those months. During the rest of the year, the overall $A_{v}$ was 95% or above.

The relationship between $A_{v}$ and impact on patient treatments is plotted for the months of January 2010 through December 2011 in (Figs. 6a)‐6(c). The treatments impacted metric includes both delayed and missed treatments and is more closely correlated with $A_{v}$ for each month ((Fig. 6c) compared to the delayed or missed treatments individually shown in (Figs. 6a)and (6b), respectively. (Figure 6b) demonstrates that with 98% $A_{v}$, it is anticipated that less than 1% of treatments will be missed. As the $A_{v}$ drops to below 96%, there is a broader range in percentage of missed treatments. For example, at an $A_{v}$ of 96%, one of the data points has fewer than 1% of missed treatments representing August 2011, while at 95% $A_{v}$, one of the data points represents nearly 4% missed treatments for February 2011. Note also that the number of missed treatments can be reduced to near zero even though $A_{v}$ is less than 100% because treatment delays are likely to occur before treatments are missed in the majority of downtime events. This is demonstrated by the data points clustered around 98% $A_{v}$. In (Fig. 6b) there are several data points corresponding to 0% missed treatments, but in (Fig. 6a) the data points corresponding to treatment delays range from 3% to 8%.

## IV. DISCUSSION

Based on the definition of $A_{v}$ in Eq. (1), the overall $A_{v}$ at IUCO for the 2011 calendar year was 96.1%, with an average of 96.9% over the last three years. MD Anderson reported a three‐year average of 97%, while LLUMC, using their “treatments missed” method, reported 98.8% in 2009.^8^, ^9^ Suzuki et al.^(8)^ calculated $A_{v}$ in a similar manner to the method reported here, with the exception that downtime was defined as any treatment break that lasted 15 minutes or longer. For our calculation of $A_{v}$, all downtime events were included, regardless of their duration. Our analysis demonstrates that delays less than 15 minutes in duration are negligible and are unlikely to cause delayed or missed treatments. Thus, excluding these minor events appears to be appropriate when calculating $A_{v}$. Our original hypothesis was that frequent 10‐minute delays would compound throughout the day, since there is no extra time scheduled between patients. Frequently, a 10‐minute delay can propagate into a longer delay if the patient must be repositioned. At our facility, this is left to the discretion of the therapist. Some patients will need to be reimaged if the break is as little as 2–3 minutes, while anesthesia patients are generally reimaged only if the treatment delay is longer than 5 minutes. Despite there being no extra time scheduled between patients, time can be saved during prostate cancer treatments. Prostate cases can routinely be delivered in less time than what is allotted, leaving 5–10 minutes of extra time that can be used as “make‐up” time for more complex cases. While shorter delay times can be an inconvenience, longer delays are more catastrophic to the patient schedule, based on our data.

The $A_{v}$ reported from LLUMC bases downtime on the fraction of missed treatments. Using this method, the average $A_{v}$ for 2011 for IUCO was calculated to be 98.3%, compared to 96.1% when calculated using Eq. (1). Of note, during May 2011 which had an $A_{v}$ of less than 88% based on Eq. (1), the $A_{v}$ using the LLUMC method was 95%. While this method is useful for quantifying the number of missed treatments, it does not reflect the fraction of time that the system is online and, thus, does not adequately reflect treatment delays. During May 2011, more than 40% of the days had downtime events where treatments were either delayed or missed. Thus, both the staff and patients were greatly inconvenienced, but the $A_{v}$ remained above 95% for the month when the calculation was performed based on the number of missed treatments.

One of the limitations of the definition of $A_{v}$ used in this study is the inclusion of scheduled beam use not directly involved with patient treatments. Ideally, to correlate $A_{v}$ with missed and delayed treatments, only scheduled beam use involving patient treatments should be included. However, $A_{v}$ during quality assurance testing is the same as during treatments, since operation and repair of the equipment is treated with the same urgency. Commissioning of new equipment and research represents less than 1% of the total time the equipment is in use and so contributes very little to the overall $A_{v}$. As a result, the decision was made to include all scheduled use in the definition of $A_{v}$ used for this study.

Investigating the data that we have collected on patient treatments and correlating with the $A_{v}$ of our facility have made us recognize the limitations of $A_{v}$ as a performance metric, particularly for patient treatments. The $A_{v}$ correlates better with treatment delays than it does with missed treatments, as shown in (Figs. 6a)and (6b). The percentage of missed treatments is highly dependent on the duration of the failures causing the decrease in $A_{v}$. Fewer treatments are missed if downtime events are frequent but of short duration. More treatments are missed in the event of shutdowns greater than four hours even though the impact on $A_{v}$ will not be as great. This is illustrated by the data in Fig. 5. There was one day during the month of December when the cyclotron was down and no patients could be treated. The $A_{v}$ for December was 95.7% and approximately 3% of patient treatments were missed, compared to August which had multiple short events during the month and had an $A_{v}$ of 96.1% with 0.4% of treatments missed. The percentage of treatment delays was similar for the two months, at approximately 6%. A similar scenario occurred during February, when there were multiple large cyclotron issues on several afternoons of the month, resulting in missed treatments. The $A_{v}$ for February was 95.2%. Based on the data in Figs. 4 and 5, it is likely that a servicing and preventive maintenance program that reduces or eliminates the occurrence of failures causing shutdowns greater than 1 hour will nearly eliminate missed treatments.

The $A_{v}$ also does not take into account when the downtime events occur, which can have a large impact on patient treatments, particularly in the pediatric population. Many pediatric patients require general anesthesia for their treatment and, consequently, are fasting prior to their treatment. If a downtime event occurs in the morning, the physician and the family must reach an agreement on the treatment for that day. An additional complicating factor is the availability of the anesthesia team depending on the length of the delay. For the pediatric population, treatment delays can easily turn into missed treatments because of the additional complications of fasting and general anesthesia for the treatment.

While the $A_{v}$ is of limited use when assessing the impact of downtime on patient treatments, it is also not a stand‐alone metric for evaluating the treatment system. While the overall $A_{v}$ can be used to determine that there is an issue with the system, additional data must be collected to determine what the issue is and how it can be improved. This is accomplished at IUCO using a log that contains the root cause of all downtime events. The cyclotron was the major contributor to downtime until 2009. After 2009, the external issues related to power failures and weather had the greatest impact on $A_{v}$. This can be verified by the MTBF which shows that while the treatment room systems have been stable for the last several years, the main issue for 2011 was power failures. The MTTR also reflects this trend with an increase in 2011 due to severe power outages. With more power failures, there are more equipment failures as well, particularly with the cyclotron, as indicated by the decrease in MTBF in 2011. With this information, PM can be focused on the area of the system where it is needed most, such as desensitizing equipment to power quality events. Also, by evaluating additional performance metrics such as the MTTR, the center can monitor its progress on diagnosing, repairing, and returning the treatment system to functional status, and preventing delays lasting longer than one hour to minimize the number of treatments that are missed.

With the shortcomings of $A_{v}$, it begs the question of whether an ideal metric for assessing the performance of a proton therapy center exists. We would argue that a perfect metric does not exist and that a combination of metrics must be used to properly capture all aspects of the functionality of a treatment facility. Along with the $A_{v}$ of the treatment system, assessment of patient treatments must be considered. It is also helpful to investigate the $A_{v}$ of the treatment system over a variety of different time durations. Yearly, and even monthly, analysis of $A_{v}$ may mask issues with the treatment system that need to be addressed, as demonstrated by the data that we present here. For example, if yearly $A_{v}$ data only is analyzed, there may be months during the year where the $A_{v}$ is low and a high number of patient treatments are adversely impacted, which would not be reflected in the yearly $A_{v}$ metric.

## V. CONCLUSIONS

With the increase in number of proton therapy centers comes the need for developing metrics to adequately assess the performance of these facilities. In this article, we demonstrated the utility of $A_{v}$ as a metric, but also showed its limitations as a stand‐alone metric. We suggest that $A_{v}$ data, as well as additional performance metrics such as the MTBF and the MTTR in conjunction with patient treatment data, be utilized when evaluating the performance of a proton therapy center.

## ACKNOWLEDGMENTS

The authors wish to acknowledge Victor Simoneaux, Chief Radiation Therapist at IUHPTC, John Kerstiens, Director of Operations and Chief Financial Officer at IUHPTC, and Thomas Meaden, Senior Cyclotron Operator at IUCO for assistance with data collection. The authors also wish to thank the reviewers of the manuscript for their comments and suggestions.
